# Stimulus–Transcription Coupling of TRPM3 Channels: A Signaling Pathway from the Plasma Membrane to the Nucleus

**DOI:** 10.3390/biom15040521

**Published:** 2025-04-02

**Authors:** Gerald Thiel, Oliver G. Rössler

**Affiliations:** Department of Medical Biochemistry and Molecular Biology, Medical Faculty, Saarland University, Building 44, 66421 Homburg, Germany; oliver.roessler@uks.eu

**Keywords:** calmodulin, calcineurin, epigenetic regulators, extracellular signal-regulated protein kinase, GPCR, phosphatidylinositol 4,5-bisphosphate, transcription factor, Zn

## Abstract

Transient receptor potential melastatin-3 (TRPM3) channels are cation channels activated by heat and chemical ligands. TRPM3 regulates heat sensation, secretion, neurotransmitter release, iris constriction, and tumor promotion. Stimulation of TRPM3 triggers an influx of Ca^2+^ ions into the cells and the initiation of an intracellular signaling cascade. TRPM3 channels are regulated by phosphatidylinositol 4,5-bisphosphate, the βγ subunit of G-protein-coupled receptors, phospholipase C, and calmodulin. Extracellular signal-regulated protein kinase ERK1/2 and c-Jun N-terminal protein kinase (JNK) function as signal transducers. The signaling cascade is negatively regulated by the protein phosphatases MKP-1 and calcineurin and increased concentrations of Zn^2+^. Stimulation of TRPM3 leads to the activation of stimulus-responsive transcription factors controlled by epigenetic regulators. Potential delayed response genes encoding the pro-inflammatory regulators interleukin-8, calcitonin gene-related peptide, and the prostaglandin-synthesizing enzyme prostaglandin endoperoxide synthase-2 have been identified. Elucidating the TRPM3-induced signaling cascade provides insights into how TRPM3 stimulation alters numerous biochemical and physiological parameters within the cell and throughout the organism and offers intervention points for manipulating TRPM3 signaling and function.

## 1. Introduction

Transient-receptor potential (TRP) channels are non-selective cation channels that have a similar modular structure but differ considerably in their primary structure [1]. TRP channels are incorporated into the membrane as tetramers and exhibit a 4-fold symmetry centered around a central ion pore. TRP channels function as sensors for a variety of stimuli, including heat and cold, as well as chemical irritants. Several plant substances have been identified as ligands for certain TRP channels, including allyl isothiocyanate, capsaicin, cinnamaldehyde, eucalyptol, hyperforin, menthol, mustard oil, and resiniferatoxin [2,3]. Mutated TRP channels have been linked to human diseases, including neurodegenerative disorders, kidney disease, pain, cancer, cardiovascular disease, and an inherited form of early-onset cataract, and are being considered as potential drug targets [2,4,5]. Several small molecule antagonists of TRP channels have already been investigated in clinical trials for pain treatment [6,7]. The largest subgroup of TRP channels is the TRPM subfamily, which includes eight members, TRPM1-TRPM8.

Here, we focus on TRPM3, a TRP channel that belongs to the thermo-TRP channels involved in temperature sensing. Several review articles have been published that addressed various aspects of TRPM3 biology, such as the regulatory role of phosphatidylinositol 4,5-bisphosphate and calmodulin, the role of TRPM3 in temperature sensing, and the specific functions of TRPM3 in the brain [8,9,10,11,12,13,14]. This review article focuses on the intracellular signaling cascade induced after stimulation of TRPM3 channels, encoded by the *TRPM3* gene on chromosome 9 of the human genome. We described TRPM3 signaling in a 2017 review article [15]. In the meantime, many new discoveries have been published, including the TRPM3 channel structure, analysis of TRPM3-associated proteins and lipids, the involvement of protein kinases and protein phosphatases as signal transducers and shut-off devices, and the identification of TRPM3-induced delayed-response genes encoding pro-inflammatory mediators. This review summarizes the new results and describes the TRPM3-induced signaling cascade from the plasma membrane to the nucleus.

## 2. Structure and Function of TRPM3 Channels

**Figure 1:**
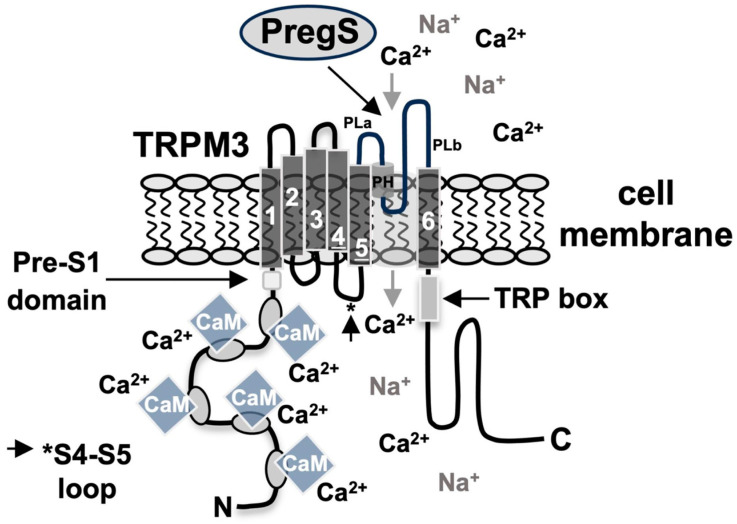
Domain structure of TRPM3 channels. TRPM3 has six transmembrane domains. The pore-forming domain is located between transmembrane regions 5 and 6. TRPM3 channels form a tetramer centered around the central pore. There are numerous putative calmodulin binding sites in the N-terminal cytoplasmic region. The proposed interaction sites of TRPM3 with phosphatidylinositol 4,5-bisphosphate include the preS1 segment (the N-terminal cytoplasmic part of TRPM3 near the first transmembrane domain), the S4-S5 linker (*) and the TRP box of the C-terminal region. The neurosteroid pregnenolone sulfate (PregS) functions as a ligand for TRPM3 channels.

TRPM3 channels have a structure typical of all TRP channels. TRPM3 has six transmembrane domains and a pore domain located between the fifth and sixth transmembrane domains (Figure 1). Four TRPM3 molecules form the central ion pore. In contrast to other TRP channels, an alternative permeation pathway distinct from the central pore has been proposed for TRPM3 channels, which are co-stimulated with pregnenolone sulfate and the antifungal agent clotrimazole [16]. This alternative permeation pathway has similarities to the omega current of Shaker K_v_ channels. However, structural data showed that the voltage-sensor-like domain of TRPM3, which is thought to form the alternative ion permeation pathway, is similar to that of other TRP channels, including conservative hydrophobic residues [17]. These data do not support the presence of an omega pore in TRPM3 and argue against the view that TRPM3 has an additional pore for ion entry [17]. In addition, analysis of the cryo-electron microscopy structure of TRPM3 revealed no evidence of an opening for ion permeation within the voltage-sensor-like domain [18]. Furthermore, it has been demonstrated that the co-application of the TRPM3 ligand pregnenolone sulfate and clotrimazole to HEK293 cells expressing TRPM3 channels almost completely blocks the signal transduction via TRPM3 channels, and TRPM3 activity induced by pregnenolone sulfate [19,20]. However, these data do not provide any evidence that clotrimazole directly inhibits TRPM3 activity. The observation that clotrimazole additionally inhibits TRPM2, TRPM8, and TRPC6 channels [19,20,21,22] suggests that this compound acts as a broad-spectrum TRP channel inhibitor. Other notable features of the TRPM3 modular structure are the TRP domain or TRP box, located on the C-terminal side of the sixth transmembrane domain, which, together with the pre-S1 segment and the S4-S5 linker, functions as a binding site for phosphatidylinositol 4,5-bisphosphate and several calmodulin binding sites within the N-terminal cytosolic portion of TRPM3.

TRPM3 is a polymodal channel that can be activated by high temperature, and noxious heat is an important physiological stimulus for TRPM3 channels. Heat activation of TRPM3 usually occurs when the temperature of the culture medium rises to 37 °C or 40 °C [23,24]. TRPM3 can also be activated by chemical stimuli. The steroid pregnenolone sulfate and the synthetic compound CIM0216 are potent chemical activators of TRPM3 channels [25,26,27,28]. Concentrations between 5 and 100 μM of pregnenolone sulfate and 1 to 20 μM of CIM0216 were employed in experiments to stimulate TRPM3 channels. Experiments with sensory neurons derived from TRPM3-deficient mice showed that TRPM3 acts as the major receptor for pregnenolone sulfate and CIM2016 [23,27]. The concentration of pregnenolone sulfate used to stimulate TRPM3 channels is in the micromolar range, suggesting that pregnenolone sulfate is more of a pharmacological agonist than a physiological ligand of TRPM3 channels [23]. Furthermore, pregnenolone sulfate is not TRPM3-specific but alters the activity of other ion channels and receptors [29,30,31,32,33]. Therefore, the involvement of TRPM3 channels after pregnenolone sulfate or CIM2016 stimulation was often confirmed using pharmacological and genetic tools [34,35,36]. The dihydropyridine compound nifedipine, known as an antagonist for L-type voltage-gated Ca^2+^ channels, has also been identified as a pharmacological activator of TRPM3 and TRPA1 channels [25,37]. However, the number of trigeminal and dorsal root ganglia neurons responding to nifedipine stimulation did not change in TRPM3-deficient mice [23], suggesting that the main target for nifedipine-induced Ca^2+^ influx is the TRPA1 channel [37].

In addition, TRPM3 activity and signaling can be inhibited by several metabolites, pharmacological inhibitors, and plant-derived compounds, including mefenamic acid, citrus fruit flavanones such as naringenin, eriodictyol, hesperetin, liquiritigenin, and isosakuranetin, and the anticonvulsant primidone [24,26,38,39]. The binding site for primidone has recently been identified [18,40]. This site overlaps with the binding site for CIM2016 but differs from the binding site for pregnenolone sulfate, as suggested earlier [24].

**Figure 2:**
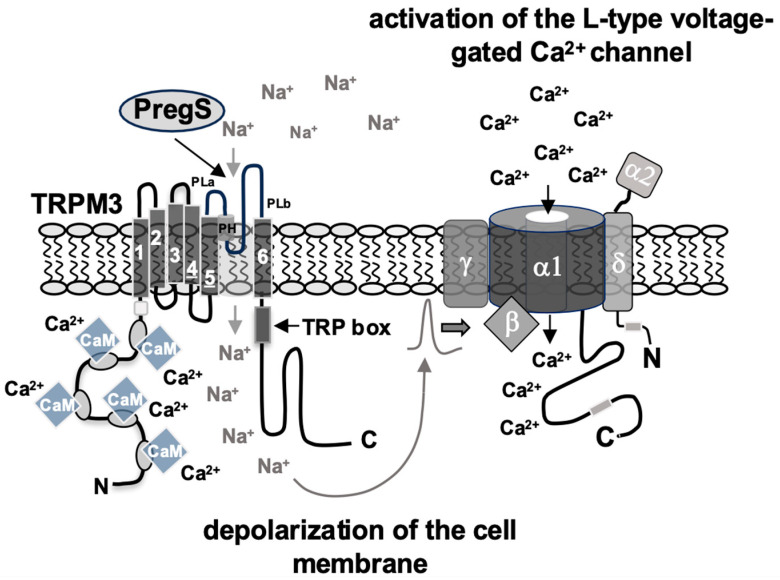
TRPM3 channels signal via activation of voltage-gated Ca^2+^ channels in insulinoma cells TRPM3 channels are non-specific cation channels that promote the influx of Ca^2+^ and Na^+^ ions into the cells upon stimulation. In insulinoma cells, stimulation of TRPM3 channels leads to an influx of Na^+^ ions into the cells, which triggers the depolarization of the plasma membrane. This activates L-type voltage-gated Ca^2+^ channels, which trigger an influx of Ca^2+^ into the cells. L-type voltage-gated Ca^2+^ channels consist of five subunits: the main α1 subunit, which forms the pore, and the auxiliary subunits α2, δ, β, and γ. The steroid pregnenolone sulfate (PregS) acts as a ligand for TRPM3 channels, interacting with the pore helix (PH) of the channel. PLa, PLb, poor loops.

Stimulation of TRPM channels induces the influx of Ca^2+^ ions into the cells, and TRPM3 has been characterized as an efficient Ca^2+^ channel [41]. Experiments with INS-1 insulinoma cells showed that TRPM3-mediated signaling is prevented by the presence of inhibitors of L-type voltage-gated Ca^2+^ channels [34]. We think that TRPM3 functions as a non-selective cation channel in this scenario, specifically as a Na^+^ channel, leading to plasma membrane depolarization and subsequent stimulation of voltage-gated Ca^2+^ channels. Activation of L-type voltage-gated Ca^2+^ channels triggers Ca^2+^ ion flux into the cells according to the ion gradient (Figure 2). The influx of Ca^2+^ into the cells, either directly through TRPM3 channels or indirectly through the activation of voltage-gated Ca^2+^ channels, and the subsequent increase in intracellular Ca^2+^ is necessary to trigger an intracellular signaling cascade [34,42]. Many TRPM3 isoforms are generated via alternative splicing and exhibit striking differences in their Ca^2+^ permeability [43]. Many experiments were carried out with cells that expressed the TRPM3 splice variant TRPM3α2 of the mouse, which has been shown to be highly permeable for Ca^2+^ ions [16,25].

TRPM3 channels are expressed in the nervous system, particularly in somatosensory neurons, adipocytes, pancreatic β-cells, kidneys, the retina, and the pituitary gland [44]. Functional experiments have shown that activation of TRPM3 channels is associated with temperature and pain-sensing, insulin and neuropeptide secretion, gene transcription, tumorigenesis, and muscle contraction [9,15]. Although TRPM3 stimulation induces insulin secretion [27], transgenic mice lacking TRPM3 show no difference in glucose-induced insulin secretion and glucose metabolism [23], suggesting that TRPM3 channels play a minor, perhaps supportive, role in pancreatic β-cell regulated glucose homeostasis. Heat sensation and the development of inflammatory heat hyperalgesia in the somatosensory system are controlled by TRPM3 channels together with TRPA1 and TRPV1 channels [23,45]. Furthermore, TRPM3 has been identified as a molecular marker for chronic fatigue syndrome/myalgic encephalomyelitis [46]. Analysis of gain-of-function mutations of TRPM3 revealed that TRPM3 plays a role in the development of neuronal disorders, including epileptic encephalopathies [9,12]. Alterations of TRPM3 are also associated with the development of autism, choroid plexus tumors, and Kabuki syndrome. A point mutation within the TRPM3 channel has been identified that causes an inherited form of early-onset cataract [5].

## 3. TRPM3 Regulation by Intracellular Signaling Proteins, Lipids, and Ions

### 3.1. TRPM3-Induced Signaling Requires Phosphatidylinositol 4,5-Bisphosphate

**Figure 3:**
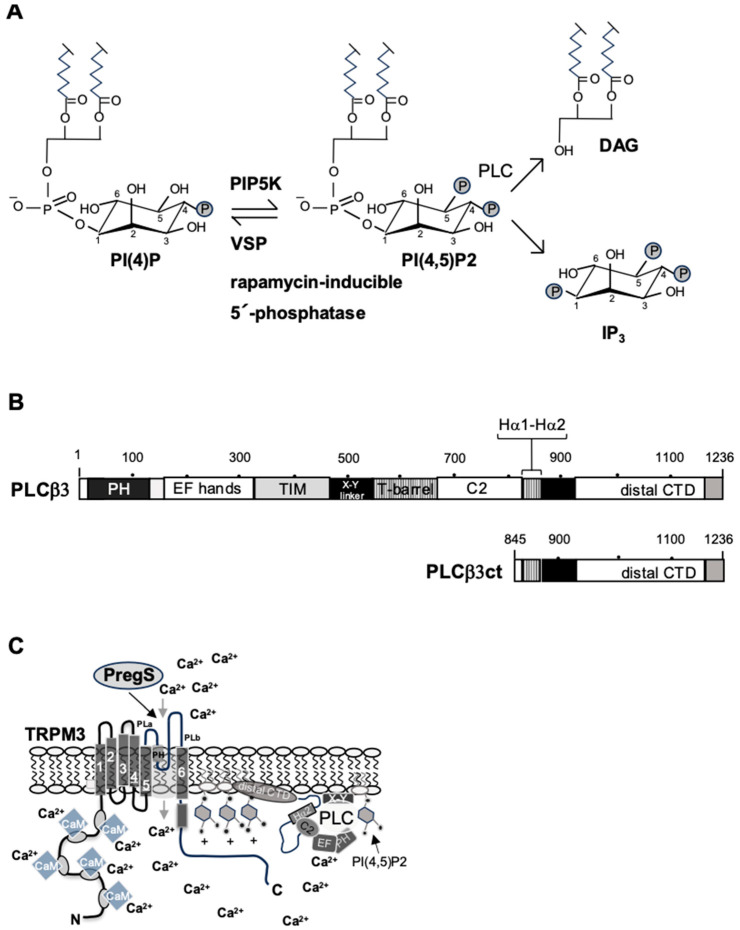
TRPM3 activity is regulated by phosphatidylinositol 4,5-bisphosphate and PLCβ3: (**A**) Biosynthesis and hydrolysis of phosphatidylinositol 4,5-bisphosphate. The phosphoinositide phosphatidylinositol 4-phosphate (PI(4)P) is converted to phosphatidylinositol 4,5-bisphosphate (PI(4,5)P2), catalyzed by phosphatidylinositol 4-phosphate 5 kinase α (PIP5K), an enzyme which catalyzes the transfer of a phosphate group to the 5′-position of the inositol ring. Expression of voltage-sensitive phosphatases (VSPs) or rapamycin-inducible 5’-phosphatases converts phosphatidylinositol 4,5-bisphosphate back to phosphatidylinositol 4-phosphate (PI(4)P). Phospholipase C (PLC) enzymes catalyze the hydrolysis of phosphatidylinositol 4,5-bisphosphate, resulting in the generation of IP_3_ and diacylglycerol (DAG). (**B**) Domain structure of phospholipase Cβ3 and the truncated variant PLCβ3ct, which contains the C-terminal domain of the enzyme. The C-terminal domain (CTD) of PLCβ enzymes consists of a proximal and a distal C-terminal domain. The proximal domain contains a helix-turn-helix motif (Hα1—Hα2), the primary binding site for Gαq, and the Hα2’ helix with autoinhibitory activity. PLCβ must interact with the membrane to hydrolyze its substrate phosphatidylinositol 4,5-bisphosphate. The distal C-terminal domain of PLCβ plays a role in membrane targeting and optimizes the orientation of the enzyme in a spatial structure that allows hydrolysis of its lipid substrate. PH, pleckstrin homology domain; TIM, catalytic triose phosphate isomerase barrel domain; X-Y linker, linker that disrupts the TIM barrel domain; EF, EF hand domain; C2, C2 domain (reproduced from Ref. [49]). (**C**) Expression of the C-terminal domain of PLCβ3 attenuates TRPM3-induced signaling. The C-terminal domain (CDT) of phospholipase Cβ harbors the primary membrane-tagging site of PLCβ3. This domain interacts with a plasma membrane target, presumably phosphatidylinositol 4,5-bisphosphate, which is also required for TRPM3 activation. The diagram shows the TRP box of TRPM3, which is involved in the binding of TRPM3 to phosphatidylinositol 4,5-bisphosphate. Thus, PLCβ and TRPM3 channels compete for the same target on the plasma membrane. PregS, pregnenolone sulfate, activates TRPM3 channels. PH, pore helix; PLa, PLb, poor loop.

It has been proposed that most TRP channels, along with other ion channels, are regulated by the lipid signaling molecule phosphatidylinositol 4,5-bisphosphate [13], a phospholipid that is highly enriched in the inner layer of the plasma membrane. Phosphatidylinositol 4,5-bisphosphate makes up about 1% of membrane phospholipids, but the concentration within the inner layer of the plasma membrane can be much higher [47]. The biosynthesis of phosphatidylinositol 4,5-bisphosphate involves the phosphorylation of phosphatidylinositol 4-phosphate [48], which is catalyzed by the enzyme phosphatidylinositol 4-phosphate 5-kinase (PIP5K). The reaction involves the transfer of a phosphate group to the D5 position of the inositol ring (Figure 3A). Phosphatidylinositol 4,5-bisphosphate is a substrate for phospholipase C (PLC) enzymes, which catalyze the hydrolysis of phosphatidylinositol 4,5-bisphosphate, generating the second messengers IP_3_ and diacylglycerol.

Various experimental strategies were used to demonstrate that TRPM3 channels are regulated by phosphatidylinositol 4,5-bisphosphate, including pharmacological and genetic methods. The most convincing results regarding the regulation of TRPM3 by phosphatidylinositol 4,5-bisphosphate were obtained with sophisticated chemical genetic and electrogenetic tools that aimed to alter the concentration of phosphatidylinositol 4,5-bisphosphate in the plasma membrane of intact cells by dephosphorylation. These tools included the voltage-activated phosphatases ci-VSP and dr-VSP as well as rapamycin-induced 4,5-phosphoinositide phosphatases, including the pseudojanin fusion protein, which consists of phosphatidylinositol 4’-phosphatase sac1 and inositol polyphosphate-5-phosphatase E (INPP5E). Expression and activation of these phosphatases in intact cells resulted in significant inhibition of TRPM3 activation [50,51]. However, the inhibition was only partial and less pronounced compared to experiments in which the regulation of TRPM8 channels by phosphatidylinositol 4,5-bisphosphate was examined so that additional regulation of TRPM3 channels by other phosphoinositides such as phosphatidylinositol 3,4,5-trisphosphat was proposed [39,40].

A pharmacological study confirmed the hypothesis that phosphatidylinositol 4,5-bisphosphate is essential for TRPM3 activation. In this study, the compound ISA-2011B was used. ISA-2011B has been shown to significantly inhibit the activity of PIP5K, the main phosphatidylinositol 4,5-bisphosphate-synthesizing enzyme, and blocks the downstream activation of the lipid kinase AKT [52,53]. Administration of ISA-2011B to cells strongly reduced TRPM3 channel-mediated signaling [54]. These data support the view that PIP5K-catalyzed biosynthesis of phosphatidylinositol 4,5-bisphosphate is essential for TRPM3 channel activation and highlight PIP5K as an important regulator of TRPM3 channel signaling through the regulation of phosphatidylinositol 4,5-bisphosphate biosynthesis.

Phosphatidylinositol 4,5-bisphosphate can directly interact with TRPM3 via hydrophobic and electrostatic interactions with specific binding sites within the channel protein. Through computer modeling and experimental analysis of mutated TRPM3 channels, three distinct binding sites for phosphatidylinositol 4,5-bisphosphate on TRPM3 were identified, including the TRP domain, the pre-S1 segment, and the S4-S5 linker [55]. Recently published structural data largely confirmed this view, showing that phosphatidylinositol 4,5-bisphosphate is found in a cavity formed by the TRP domain, the pre-S1 helices, and the S4-S5 linker [17].

### 3.2. Inhibition of TRPM3 Signaling by the βγ Subunits of Trimeric G Proteins

Stimulation of the Gαq-coupled M1 muscarinic acetylcholine receptor was shown to impair activation of TRPM3 [50,51]. TRPM3 inhibition was also observed following activation of Gαi/o or Gαs-coupled receptors [56], leading to the characterization of TRPM3 as a “GPCR-inhibited ion channel” [17]. Genetic and pharmacological experiments have shown that this inhibition occurs through released Gβγ subunits of trimeric G proteins following receptor stimulation [57,58,59]. TRPM3 channels are not regulated by the α-subunit of Gq [54,59], in contrast to direct binding of the α-subunit of Gq to TRPM8 channels [60,61]. Furthermore, the expression of the regulator of G-protein signaling-2 (RGS2), which stimulates the GTPase activity of Gαq and inactivates Gαq, strongly inhibits TRPM8 signaling but has no effect on the TRPM3-induced signaling cascade [54].

Alternative splicing gives rise to many different TRPM3 isoforms, including two variants, TRPM3α4 and TRPM3α5, that do not respond to inhibition by Gβγ. Comparison of the primary structure of these variants with those that respond to Gβγ revealed the absence of 10 amino acids encoded by exon 17, suggesting that translation of this exon acts as a negative control for Gβγ regulation of TRPM3 channels. Biochemical analysis, mutagenesis studies, and a crystal structure analysis of Gβγ with the TRPM3 peptide support the view that this 10-amino acid sequence is the binding site for Gβγ at TRPM3 channels [62]. Electrophysiological recordings of a C-terminal truncated TRPM3 protein with a prenylation-deficient Gβγ showed a high binding affinity of the truncated TRPM3 channel for Gβγ in membrane patches. However, a structural analysis of TRPM3 in the presence of Gβγ showed that Gβγ is rather loosely bound to TRPM3 and that an artificially high concentration of Gβγ was required to show complexes between TRPM3 and Gβγ that could be expected based on the electrophysiological recordings [17]. The authors concluded from these data that important components were missing from their cryo-electron microscopy study. One of these missing components could be phosphatidylinositol 4,5-bisphosphate, which may be required to stabilize the interaction between TRPM3 and Gβγ [17].

### 3.3. Phospholipase C Negatively Affects TRPM3 Signaling

Phospholipase C (PLC) enzymes catalyze the hydrolysis of phosphatidylinositol 4,5-bisphosphate, resulting in the formation of 1,4,5-triphosphate (IP_3_) and diacylglycerol (DAG). Activation of PLC, therefore, decreases the concentration of phosphatidylinositol 4,5-bisphosphate required for TRPM3-induced signaling. The observation that stimulation of the Gαq-coupled M1 muscarinic acetylcholine receptor, which results in a stimulation of PLC, impairs the activation of TRPM3 [50,51] led to the hypothesis that activation of the Gαq-coupled receptor activation inhibits TRPM3 by reducing the concentration of phosphatidylinositol 4,5-bisphosphate. This hypothesis was checked with indirect measurements of membrane-bound phosphatidylinositol 4,5-bisphosphate, involving assays that determine the translocation PLCγ-PH domain or similar phosphatidylinositol 4,5-bisphosphate-binding domains from the plasma membrane to the cytoplasm. However, these assays have many problems and disadvantages; they are not completely specific for phosphatidylinositol 4,5-bisphosphate and should be treated with caution, as has been recently discussed [54,63].

In general, it is difficult to quantify the reduction in phosphatidylinositol 4,5-bisphosphate concentration after PLC activation because the time period until resynthesis from phosphatidylinositol 4-phosphate, which restores the initial phosphatidylinositol 4,5-bisphosphate concentration, may be short [64,65]. Therefore, in most cases, there is no significant measurable depletion of phosphatidylinositol 4,5-bisphosphate concentration after PLC activation [47]. Stimulation of the Gαq-coupled receptor releases Gα and Gβγ subunits, both of which independently bind to PLCβ and affect its activity. Gαq increases the *k_cat_* of PLCβ by altering PLCβ autoinhibition, mediated by the X-Y linker, and by promoting alignment of the catalytic core of the enzyme with the membrane. The Gβγ subunits activate phosphatidylinositol 4,5-bisphosphate hydrolysis by relocating PLCβ closer to the membrane, i.e., to its lipid substrate [66,67].

Recently, we discovered that the C-terminal domains of PLCβ1 and PLCβ3 interact with plasma membrane targets and block the biological activation of TRPM3 channels [49] (Figure 3B,C). This target is likely to be phosphatidylinositol 4,5-bisphosphate since about two-thirds of the phosphatidylinositol 4,5-bisphosphate pool is known to be sequestered by binding proteins. This pool is not freely available to effector proteins that require phosphatidylinositol 4,5-bisphosphate [68]. Thus, PLCβ inhibits TRPM3 activation via its C-terminal domain even in the absence of stimulation.

### 3.4. Calmodulin Is Required for TRPM3 Signaling

Calmodulin, a small acidic Ca^2+^ binding protein, controls the activity of numerous enzymes, including the Ca^2+^/calmodulin-dependent protein phosphatase calcineurin, and regulates the activities of several ion channels, including several TRPM channels [11]. Several calmodulin binding sites have been mapped within the TRPM3 molecule. In vitro binding assays identified two calmodulin binding sites in the N-terminal region of TRPM3 [69]. Based on a prediction in silico, a combination of pull-down and dot blot analyses led to the identification of five calmodulin binding sites within the amino-terminal region of TRPM3 [70], two of which (CaMBS1 and 3) overlap with the originally proposed calmodulin binding sites. It is noteworthy that the proposed calmodulin binding sites CaMBS2 and 3 are present in all TRPM3 splice variants, whereas binding sites CaMBS1, 4, and 5 are subject to alternative splicing and are, therefore, only found in some splice variants of TRPM3 [70].

Pharmacological and genetic experiments have shown that calmodulin is a positive regulator of TRPM3 channels and is required for intracellular signaling following stimulation of TRPM3 channels [71]. In particular, expression of a calmodulin mutant that was unable to bind Ca^2+^ and therefore resembled the Ca^2+^-free form of calmodulin (apoCaM) impaired intracellular signaling and gene transcription after stimulation of TRPM3 channels, suggesting that calmodulin is a positive regulator of TRPM3 channels. In vitro experiments with calmodulin-Sepharose columns showed that Ca^2+^-bound calmodulin was bound to TRPM3. Furthermore, residual binding of Ca^2+^-free calmodulin was observed in pull-down experiments [70]. We, therefore, assume that calmodulin is associated with the TRPM3 channel even before the increase in intracellular Ca^2+^ concentration. Ca^2+^ ions then bind to the pre-associated calmodulin and promote the activation of the TRPM3 channel. Accordingly, the calmodulin mutant binds to TRPM3, competes with wild-type calmodulin, and, therefore, has a dominant-negative effect. A similar scenario has been described for the regulation of voltage-gated Ca^2+^ channels [72,73,74,75,76,77] and Ca^2+^-activated Cl^-^ and K^+^ channels [78,79]. Identifying which of the putative calmodulin binding sites is essential for the regulation of the TRPM3 channel activity by calmodulin remains to be determined.

It has also been suggested that the binding of calmodulin to a specific site within the TRPM3 molecule is essential for TRPM3 activity based on experiments conducted with a mutated TRPM3 channel that lacks one of these binding sites. However, not only was the cytosolic Ca^2+^ concentration reduced in pregnenolone sulfate-stimulated cells expressing the mutated TRPM3 protein, but the overall expression was greatly reduced compared to the intact channel [70], suggesting that the low Ca^2+^ signal obtained after stimulation of the mutated TRPM3 channel was a consequence of its reduced expression levels. Furthermore, the expression levels of the mutated channel at the plasma membrane were not measured, although it is known from experiments with other TRP channels that the amount of functional TRP channels at the plasma membrane can be very low after overexpression [41]. It would be interesting to know whether the deletion of a putative calmodulin bind site is responsible for the reduced expression levels of this mutated TRM3 channel.

### 3.5. Zn^2+^ Ions Negatively Regulate TRPM3 Signaling

Zn^2+^ ions enter cells via Zn^2+^ transporters or Ca^2+^ channels and influence the structure and activity of many intracellular proteins. It has been suggested that Zn^2+^ ions could use TRPM3 channels to enter the cells [80]. However, this assumption was based on experiments conducted with highly toxic concentrations of Zn^2+^ ions. Secretory granules of pancreatic β-cells or synaptic vesicles contain Zn^2+^ ions, which are released together with insulin or glutamate, respectively. Recently, it has been shown that the application of Zn^2+^ ions to the cells reduces the intracellular signaling induced by stimulation of TRPM3 channels and voltage-gated calcium channels, respectively [81]. We propose that Zn^2+^ ions released after stimulation of pancreatic β-cells or neurons, for example, act as negative feedback on exocytosis. Although it has been proposed that Zn^2+^ ions may act as second messengers in cells, Zn^2+^ ions cannot replace Ca^2+^ ions to trigger an intracellular signaling cascade after stimulation of TRPM3 or voltage-gated Ca^2+^ channels.

We think that Zn^2+^ ions bind to signaling molecules in the cells that are required for Ca^2+^-mediated signaling from the channels to the nucleus, thus disrupting the TRPM3-induced intracellular signaling cascade. Zn^2+^ ions have been shown to interfere with the leptin and insulin signaling pathway by targeting protein tyrosine phosphatase 1B [82]. Protein kinase C (PKC) may also be an intracellular target for Zn^2+^ since Zn^2+^ ions have been shown to impair protein kinase C-induced nuclear signaling [81]. Zn^2+^ ions could also directly inhibit Ca^2+^ influx through Ca^2+^ channels, as suggested [83,84].

## 4. Stimulus-Responsive Protein Kinases Act as Signaling Transducers Within the TRPM3 Induced Signaling Cascade

The fact that the influx of Ca^2+^ ions into the cells is crucial for triggering a signaling cascade after stimulation of TRPM3 channels points to the search for Ca^2+^-regulated signaling molecules. Intracellular targets of Ca^2+^ ions are the PKC isoenzymes, which are among the most important regulators of intracellular signaling and include several Ca^2+^-dependent isoforms. Pharmacological inhibition of PKC has been shown to inhibit the TRPM3-induced signaling pathway [81].

**Figure 4:**
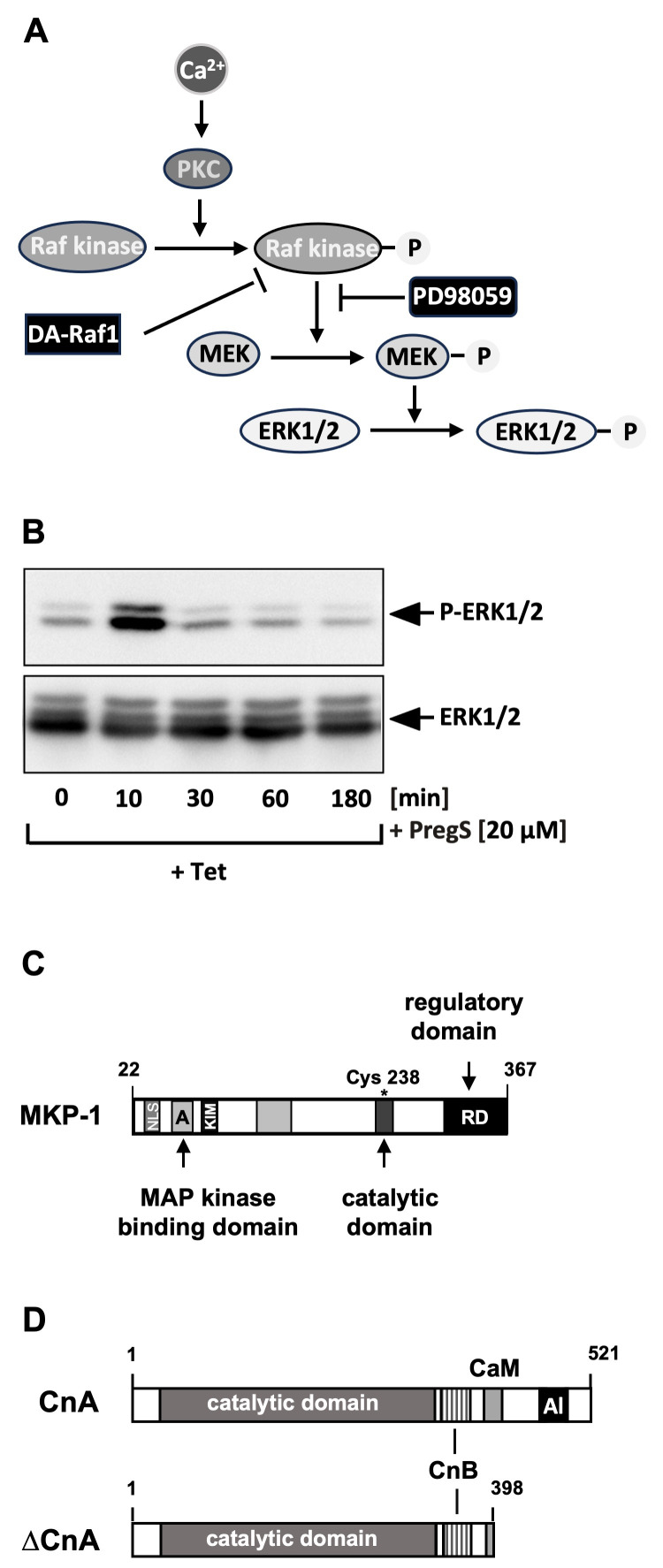
TRPM3 signaling is regulated by protein kinases and phosphatases: (**A**) Activation of TRPM3 channels stimulates the Raf/MEK/ERK signaling pathway. Activation of TRPM3 channels results in an influx of Ca^2+^ ions into the cytosol. Ca^2+^ activates Ca^2+^-dependent isoforms of protein kinase C (PKC), which leads to an activation of Raf kinase. This protein kinase activates the mitogen-activated protein kinase (MAP) kinase (MEK) by phosphorylation, and phosphorylated MEK phosphorylates and activates extracellular signal-regulated protein kinase ERK1/2. Raf kinase activity is compromised by the expression of DA-Raf1, a dominant-negative form of A-Raf protein kinase. The compound PD98059 prevents the phosphorylation of MEK by Raf kinase. (**B**) TRPM3 channel stimulation with pregnenolone sulfate (PregS) induces the transient phosphorylation of ERK1/2. HEK293 cells containing a tetracycline-inducible TRPM3 transcription unit were serum-starved for 24 h in the presence of tetracycline to induce TRPM3 expression and then stimulated with pregnenolone sulfate to activate TRPM3 expression. Shown is a Western blot analysis performed with a monoclonal antibody against the phosphorylated active form of ERK. An antibody detecting ERK1/2 was used as loading control (reproduced from Ref. [87] with kind permission from Wiley Periodicals, Inc., Hoboken, NJ, USA). (**C**) Modular structure of MAP kinase phosphatase-1 (MKP-1). The enzyme dephosphorylates and inactivates the MAP kinases ERK1/2, p38 protein kinase, and JNK in the nucleus. The N-terminus contains the nuclear localization signal and the substrate binding site. The C-terminus contains the catalytic domain, which catalyzes the dephosphorylation of tyrosine/threonine residues of its substrates. Cysteine residue 258 is essential for enzymatic activity. Regulatory domains responsible for stability and proteasomal degradation are present at the extreme C-terminus. (**D**) Domain structure of calcineurin A and the truncated constitutively active mutant ΔCnA. The binding sites for calcineurin B (CnB) and calmodulin (CaM) are shown, as well as the C-terminal autoinhibitory domain (AI).

Numerous substrates are known for the various isoforms of PKC. One of these major effector signaling pathways of PKC is the Raf/MEK/ERK signaling pathway, which consists of the protein kinases Raf, mitogen-activated protein kinase kinase (MEK), and extracellular signal-regulated protein kinase (ERK). Each kinase is activated by phosphorylation, as shown in Figure 4A. The involvement of Raf in the TRPM3-induced signaling cascade was demonstrated in expression experiments of a dominant-negative form of A-Raf, one of the Raf kinase isoforms [34]. A major substrate for Raf is the mitogen-activated protein kinase (MAP) kinase (MEK) [85,86]. Pharmacological inhibition of Raf-mediated MEK phosphorylation blocks the signaling cascade triggered by stimulation of TRPM3 channels [34,87]. Phosphorylated MEK is active and phosphorylates the protein kinase extracellular signal-regulated protein kinase (ERK1/2) [88] that plays an essential role as a signal transducer from the cytoplasm to the nucleus [89,90]. Phosphorylated ERK1/2 can easily detected with phospho-specific antibodies, thus providing a biochemical test to detect stimulated TRPM3 channels—in addition to measuring intracellular Ca^2+^ concentrations or transmembrane currents. Figure 4B shows that ERK1/2 is rapidly and transiently phosphorylated upon stimulation of TRPM3 channels with its ligand pregnenolone sulfate. Knockdown experiments in which small hairpin RNAs specific for the ERK1 and ERK2 isoforms were expressed confirmed the important role of ERK1/2 in TRPM3-induced signaling and gene transcription [87]. These data were further confirmed by the observation that the expression of a gain-of-function mutant of TRPM3 led to increased levels of phosphorylated ERK1/2 in the lens [91]. In this context, it would be interesting to know whether stimulation of the proposed alternative ion permeation pathway of TRPM3 could also trigger the phosphorylation of ERK1/2 and thus induce a signaling cascade like that of ion flux through the central pore of TRPM3.

Furthermore, experiments using RNAi technology suggest that c-Jun N-terminal protein kinase (JNK) is also part of the TRPM3-induced signaling cascade [87] and acts as a signal transducer. This view is supported by the observation that overexpression of MAP kinase phosphatase-5 (MKP-5), a protein phosphatase that dephosphorylates and inactivates JNK (and also p38 protein kinase), but not ERK1/2 [92], attenuates TRPM3-induced activation of the transcription factor AP-1 [87]. The signaling cascade connecting TRPM3 channels and JNK activation remains to be elucidated.

## 5. Protein Phosphatases Act as Shut-Off Devices of the TRPM3-Induced Signaling Cascade

The phosphorylated and activated protein kinase ERK1/2 translocates into the nucleus and alters the gene expression pattern by phosphorylating gene regulatory proteins. This signaling pathway is attenuated by nuclear MAP kinase phosphatases, which dephosphorylates and inactivate the protein kinases ERK1/2, JNK, and the protein kinase p38 [93,94]. In particular, expression of the nuclear MAP kinase phosphatase-1 (MKP-1) (Figure 4C) interrupts the signaling cascade initiated by TRPM3 stimulation [34,42,95], suggesting that MKP-1 functions as a nuclear shut-off device by catalyzing the dephosphorylation and thus the inactivation of nuclear ERK1/2 and JNK. Expression experiments using a JNK-specific shRNA and overexpression experiments of MKP-5, which dephosphorylates JNK and p38 protein kinase, support the view that JNK—in addition to ERK1/2—acts as a signal transducer of TRPM3 channels.

The influx of Ca^2+^ ions into the cells activates not only PKC but also calcineurin, a Ca^2+^ and calmodulin-dependent protein phosphatase [96]. The calcineurin holoenzyme is a heterodimer consisting of the catalytic calcineurin A subunit (CnA) (Figure 4D) and a tightly bound regulatory B subunit (CnB), that shares homology with calmodulin and also binds Ca^2+^ ions via EF-hand Ca^2+^ binding motifs. Calcineurin is inactive at low Ca^2+^ concentrations due to the interaction of the autoinhibitory domain with the catalytic center. An influx of Ca^2+^ ions into the cytosol activates calcineurin through the binding of a Ca^2+^/calmodulin complex to calcineurin A, displacing the autoinhibitory domain from the catalytic site [97,98].

A constitutively active form of calcineurin is generated by deleting the autoinhibitory domain of calcineurin A and the binding sites for the calcineurin B subunit (CnB) and calmodulin. Gene transcription induced by stimulation of TRPM3 channels is significantly attenuated in cells expressing this calcineurin A mutant [34,87], suggesting that calcineurin is—similar to MKP-1 and MKP-5—part of a negative feedback loop that inhibits the TRPM3-induced signaling pathway by dephosphorylating certain substrates. One of the substrates of calcineurin is the ternary complex factor Elk-1 [99,100,101], a transcription factor that serves as a nuclear target of the TRPM3-induced signaling cascade and regulates the expression and activity of the transcription factors Egr-1 and AP-1.

Calmodulin is essential for the activation of calcineurin since only the binding of a Ca^2+^/calmodulin complex to CnA activates the holoenzyme. Calmodulin is also necessary for the TRPM3 signaling cascade to proceed following the influx of Ca^2+^ ions as a result of TRPM3 stimulation. Calmodulin, therefore, plays a dual role in regulating TRPM3 signaling: Calmodulin is required for the activity of TRPM3, probably through direct binding to the channel. Calmodulin also activates calcineurin, which acts as a shut-off device for TRPM3 signaling [71].

## 6. TRPM3 Stimulation Leads to the Activation of Stimulus-Responsive Transcription Factors

The translocation of the activated protein kinases ERK1/2 and JNK transports the signal triggered by the stimulation of TRPM3 channels into the nucleus, where the activity of gene regulatory proteins is altered by phosphorylation. These stimulus-responsive transcription factors are transiently activated by either inducing their biosynthesis and/or regulating their activity through phosphorylation. They bind to the regulatory regions of delayed response genes and stimulate their transcription. The gene products of these delayed response genes are then responsible for the biochemical and physiological changes observed as a result of the stimulation. The identification of delayed response genes within the TRPM3 signaling cascade is, therefore, an important task now and in the future.

### 6.1. Egr-1, Elk-1

**Figure 5:**
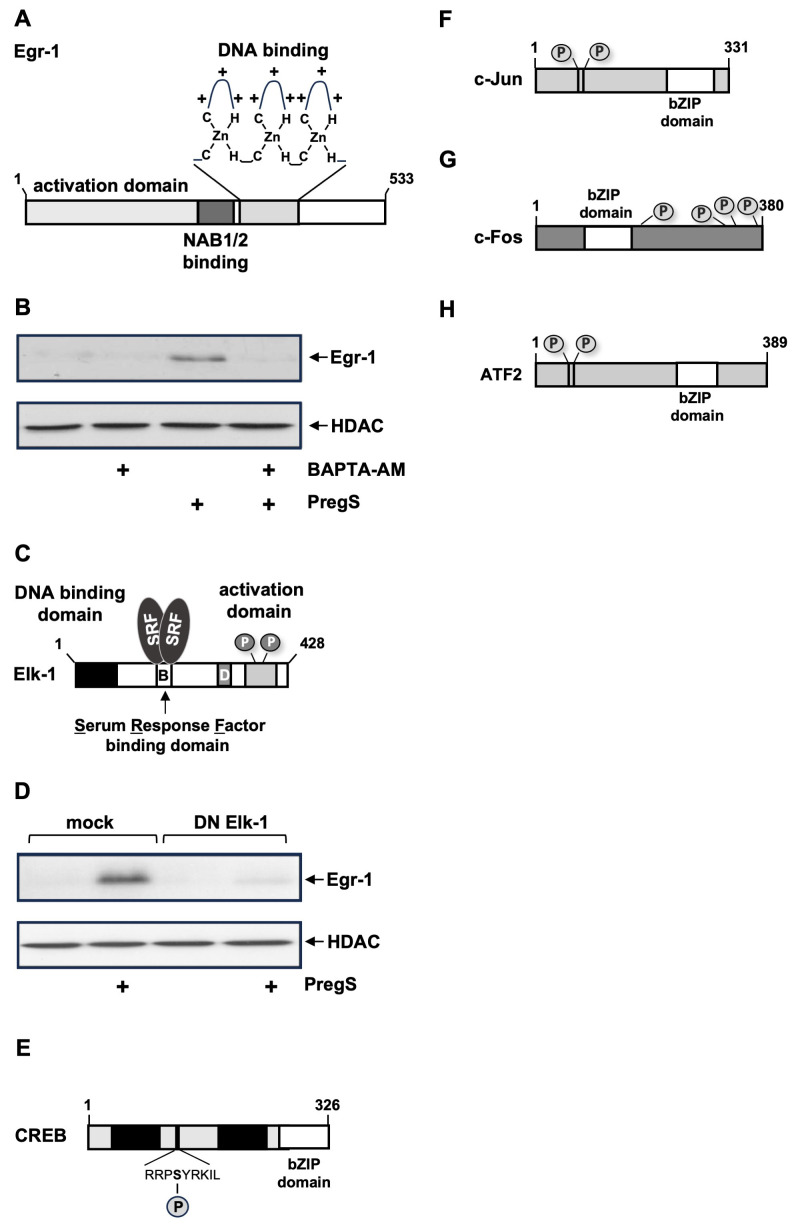
TRPM3 stimulation results in the activation of stimulus-responsive transcription factors: (**A**) Modular structure of Egr-1. Egr-1 has a cluster of three zinc finger motifs that function as a DNA binding domain. Each zinc finger contains a short antiparallel β sheet and an α helix in the Egr-1 molecule, which together form a globular structure held together by hydrophobic residues and the zinc ion. Two cysteine residues (C) derived from the β sheet and two histidine residues (H) derived from the α helix coordinate the zinc ion. The zinc fingers wrap around the DNA. The α helices fit into the major groove. The N-terminus contains an extensive transcriptional activation domain. Egr-1 activity is negatively regulated by the co-repressor proteins NAB1 and NAB2, which bind directly to the Egr-1 molecule at a site between the activation domain and the DNA binding domain. (**B**) Stimulation of INS-1 insulinoma cells with the TRPM3 ligand pregnenolone sulfate (PregS) leads to the biosynthesis of Egr-1, which requires an increase in intracellular Ca^2+^. INS-1 cells were preincubated for 1 h with the Ca^2+^ chelator BAPTA-AM (25 μM) and then stimulated with pregnenolone sulfate (PregS, 50 μM). A Western blot analysis developed with an anti-Egr-1 antibody is shown. The anti-histone deacetylase-1 (HDAC1) antibody was used as a loading control (reproduced from Ref. [34]). (**C**) Modular organization of the transcription factor Elk-1. Elk-1 is a ternary complex factor that binds to the serum-response element (SRE) together with a dimer of the serum response factor (SRF). The DNA binding domain is localized at the N-terminus of Elk-1, and the transcriptional activation domain is localized on the C-terminus. The B domain is required for the formation of the ternary Elk-1-SRF complex. The transcriptional activity of Elk-1 is regulated by phosphorylation. (**D**) Elk-1 is essential for TRPM3-induced biosynthesis of Egr-1. INS-1 insulinoma cells were stimulated with pregnenolone sulfate in the absence of a dominant negative mutant of Elk-1 (DN Elk-1). A Western blot analysis is shown. The blot was developed with an antibody directed against Egr-1. The anti-HDAC1 antibody was used as a loading control (reproduced from Ref. [34]). (**E**–**H**) Modular structure of the TRPM3-activatable bZIP transcription factors CREB (**E**), c-Jun (**F**), c-Fos (**G**), and ATF2 (**H**). The bZIP domains and the phosphorylation sites are shown.

The first transcription factor identified as being TRPM3-responsive was the zinc finger protein Egr-1 (Figure 5A), which is regulated by its biosynthesis. Figure 5B shows that stimulation of TRPM3 channels with the TRPM3 ligand pregnenolone sulfate activates the biosynthesis of Egr-1 in insulinoma cells, a process that does not occur when the increase in intracellular Ca^2+^ concentration is pharmacologically prevented [26,34]. TRPM3 stimulation has been shown to increase the concentration of biologically active Egr-1 [26]. Egr-1 regulates the expression of the homeobox protein Pdx-1 in pancreatic β-cells, a major regulator of insulin gene transcription. Thus, increased insulin mRNA levels were detected in pregnenolone sulfate-stimulated insulinoma cells. Egr-1 has also been shown to regulate the expression of the mitogen basic fibroblast growth factor [34], thereby linking TRPM3 stimulation to cell growth.

The major regulator of Egr-1 gene transcription is the ternary complex factor Elk-1 (Figure 5C), which binds to five serum response elements (SRE) within the Egr-1 proximal promoter region. Elk-1 is activated upon stimulation of TRPM3 channels with pregnenolone sulfate [26,102]. Figure 5D shows that the expression of a dominant-negative mutant of Elk-1 in insulinoma cells prevents TRPM3-induced Egr-1 biosynthesis. Likewise, Elk-1 is responsible for the upregulation of c-Fos expression after TRPM3 stimulation [103]. Expression of a dominant-negative mutant of Elk-1 blocks the expression of c-Fos after stimulation of TRPM3. c-Fos is a transcription factor that, together with other basic region leucine zipper (bZIP) proteins, constitutes the AP-1 transcription factor complex.

Elk-1 acts as a master regulator of stimulus-induced gene transcription. Under basal conditions, Elk-1 is in an inactive state due to the binding of a SUMO-histone deacetylase complex. Stimulation of MAP kinases, including ERK1/2 and JNK, activates Elk-1 by phosphorylation, while subsequent dephosphorylation, catalyzed by calcineurin, facilitates the re-SUMOylation of Elk-1, which returns Elk-1 to a transcriptionally inactive state [101]. Stimulation of TRPM3 channels with pregnenolone sulfate increases the transcriptional activation potential of Elk-1, involving a rise in intracellular Ca^2+^ and activation of ERK1/2. This signaling pathway is prevented in cells expressing MKP-1 or a constitutively active form of calcineurin A [102]. Both phosphatases act as a negative feedback loop in the signaling cascade that links TRPM3 stimulation with Elk-1 activation.

Gene knockout experiments showed that TRPM3 is not required for the regulation of basal glucose homeostasis [23]. In contrast, the TRPM3-induced transcription factors Egr-1 and Elk-1 are essential for the regulation of glucose homeostasis in transgenic mice models [104,105]. Furthermore, these mice showed a striking reduction in islet size. We propose that TRPM3 plays a supportive role in pancreatic β-cells. TRPM3 stimulation depolarizes the plasma membrane, thus activating voltage-gated Ca^2+^ channels, leading to an influx of Ca^2+^ ions in the cells and subsequent exocytosis of insulin. TRPM3 stimulation also activates the transcription factors Egr-1 and Elk-1, which stimulate insulin biosynthesis via Pdx-1 and ensure that the islets are large enough to synthesize sufficient insulin.

### 6.2. Basic Region Leucine Zipper (bZIP) Transcription Factors

TRPM3 stimulation activates several bZIP proteins, which form dimers according to a specific dimerization code [106]. The leucine zipper is responsible for dimerization and the basic region for DNA binding. The bZIP proteins CREB, c-Fos, c-Jun, and ATF2 are phosphoproteins, and phosphorylation is required for their activation.

The transcription factor CREB (cyclic AMP-response element binding protein) (Figure 5E) is a major activator of cAMP and Ca^2+^-induced transcription and acts as a master integrator of numerous signaling pathways induced by hormones, neurotransmitters, metabolites, and neurotrophins. The cognate DNA binding site is termed the cAMP response element (CRE) and comprises the sequence 5′ -TGACGTCA-3′. Several protein kinases phosphorylate CREB, including the cAMP-dependent protein kinase PKA, the Ca^2+^/calmodulin-dependent protein kinase CaMKIV, and the ERK1/2-activated mitogen and stress-activated protein kinase MSK. CREB is phosphorylated in TRPM3-expressing insulinoma cells that have been stimulated with pregnenolone sulfate. Furthermore, CRE-regulated gene transcription is upregulated as a result of TRPM3 channel stimulation [35,107].

The intracellular signaling cascade induced by the stimulation of TRPM3 channels is connected to the nucleus via the signal transducer protein kinases ERK1/2 and JNK. This activates the transcription factor AP-1 (activator protein-1) [35]. AP-1 consists of a dimer of the bZIP proteins, which originates from the transcription factor families Fos, Jun, and ATF. A major nuclear substrate for JNK is the transcription factor c-Jun (Figure 5F). In addition, c-Jun is a target of the ERK1/2 signaling pathway [108,109]. c-Jun is a major component of the AP-1 transcription factor. Phosphorylation of c-Jun is essential for the upregulation of the transcriptional activation potential of c-Jun. Stimulation of TRPM3 in insulinoma cells results in the phosphorylation of c-Jun, indicating that c-Jun is activated following TRPM3 stimulation [35]. c-Jun binds to its own promoter via two AP-1 binding sites. This explains the fact that TRPM3 stimulation induces an upregulation of c-Jun levels, which occurs with a time delay after phosphorylation and activation of c-Jun [35,87]. The modular structure of c-Fos shows a central bZIP domain, a C-terminal transactivation domain, and numerous phosphorylation sites (Figure 5G). c-Fos is frequently found as a component of AP-1. Stimulation of TRPM3 channels in insulinoma cells leads to the upregulation of c-Fos promoter activity and an increase in c-Fos biosynthesis [35]. TRPM3-induced expression of c-Fos requires CREB, AP-1, and Elk-1, which bind to distinct sites within the proximal c-Fos promoter [103]. Experiments in which either an ATF2-specific shRNA or a dominant-negative mutant of ATF2 was expressed showed that the bZIP protein ATF2 is also part of the signaling cascade that begins with the stimulation of TRPM3 channels and leads to the activation of AP-1 [42]. The modular structure of ATF2 reveals a C-terminal bZIP domain and two phosphorylation sites essential for its activation.

### 6.3. Specificity of TRPM3-Induced Gene Transcription

The fact that TRPM3 signaling results in the activation of several stimulus-responsive transcription factors could suggest that TRPM3 stimulation is non-specifically linked to signaling cascades within the cells. However, the fact that TRPM3 stimulation does not activate NF-κB and Nrf-2 indicates that TRPM3 selectively stimulates certain stimulus-responsive transcription factors [26,95].

## 7. Activation of Delayed-Response Genes After Stimulation of TRPM3 Channels

The transient activation of stimulus-responsive transcription factors triggers a secondary wave of delayed-response gene transcription. Identification of these genes will help to elucidate the biological functions of TRPM3. TRPM3 stimulation induces the secretion of calcitonin-gene-related peptide (CGRP) in skin nerve terminals stimulated with pregnenolone sulfate [27]. CGRP is a neuropeptide synthesized and released by nociceptors and acts as a neuromodulator inducing local vascular and inflammatory effects. An analysis of the TRPV1-induced signaling pathway revealed that stimulation of TRPV1 induces the expression of CGRP through the transcription factor CREB, which interacts with a conserved CRE (sequence 5′-TGACGTCA-3′) in the CGRP promoter region [110]. Therefore, it is likely that TRPM3 channel stimulation also activates CGRP gene expression via CREB. The gene encoding the pro-inflammatory cytokine interleukin-8 (IL-8), also known as CXCL8, was identified as a delayed-response gene to TRPM3 stimulation [95]. IL-8 acts as a chemoattractant and neutrophil activator, and expression and secretion of IL-8 have been associated with inflammatory disorders. Furthermore, the prostaglandin endoperoxide synthase-2 gene promoter has recently been shown to be activated after stimulation of TRPM3 channels [Brandmeier and Thiel, manuscript in preparation]. This enzyme catalyzes the rate-limiting reaction in prostaglandin biosynthesis. Prostaglandins are critical mediators of infection and inflammation. Thus, stimulation of TRPM3 activates the biosynthesis of three different pro-inflammatory mediators, pointing to a direction for future research on the biological function of TRPM3 channels.

## 8. TRPM3-Induced Activation of Transcription Is Controlled by Epigenetic Regulators

Transcription mediated by TRPM3 channel stimulation is not only dependent on the activity of sequence-specific transcription factors that bind to their cognate sites in the promoter region of target genes. Transcriptional activation also depends on the chromatin architecture of target genes. DNA in chromatin is complexed with histone proteins, and these complexes may be compact, preventing transcription factors and RNA polymerase II from binding to DNA. As a result, no transcription takes place. Chromatin can also exist in an open configuration, which allows for the recruitment of RNA polymerase II and makes the DNA accessible for transcription factors. Transcription factors interact with numerous coregulator proteins, which are chromatin-modifying enzymes or subunits of ATP-dependent protein complexes that affect the chromatin status. The structure of the chromatin is determined by the activity of enzymes that add or remove post-translational modifications to histone proteins.

Histone acetyltransferases catalyze the acetylation of lysine residues of histone proteins (and non-histone proteins). This post-translational modification neutralizes the positive charges of lysine residues of histones and thus weakens the interaction between histone proteins and the acidic DNA. The chromatin converts to an open configuration that facilitates the binding of transcription factors to DNA [111]. The acetyltransferases CBP (CREB-binding protein) and p300 function as transcriptional co-activator proteins for transcription factors that are activated after TRPM3 stimulation, including CREB, c-Jun, Egr-1, and Elk-1 [112,113,114,115]. Pharmacological inhibition of CBP/p300 attenuates the activation of AP-1 and CREB-regulated gene transcription and reduces the transcriptional activation potential of Elk-1 and c-Fos after stimulation of TRPM3 channels. Furthermore, transcription of the AP-1 target gene IL-8 was significantly reduced in cells treated with the CBP/p300 inhibitor [107]. Together, these data support the view that the acetyltransferases CBP and p300 regulate TRPM3-induced transcription at the epigenetic level.

Histone acetyltransferases are “writers” of post-translational histone modifications by catalyzing the acetylation of lysine residues of histone proteins. These marks are recognized by bromodomain-containing proteins, the “readers”, which bind to acetylated lysine residues via a central hydrophobic pocket [116]. Using a pan-BET protein inhibitor, it was shown that inhibition of bromodomain and extra terminal domain (BET) protein attenuates TRPM3-induced activation of AP-1 and CREB and enhancement of the transcriptional activation potential of c-Fos and Elk-1. It has also been shown that TRPM3-induced transcription of the IL-8 gene is controlled by BET proteins [107]. These data are supported by the observation that TRPM3-induced genes encoding IL-8, c-Fos, and prostaglandin endoperoxide synthase-2 are occupied by BET proteins [117,118].

## 9. Conclusions and Future Prospects

**Figure 6:**
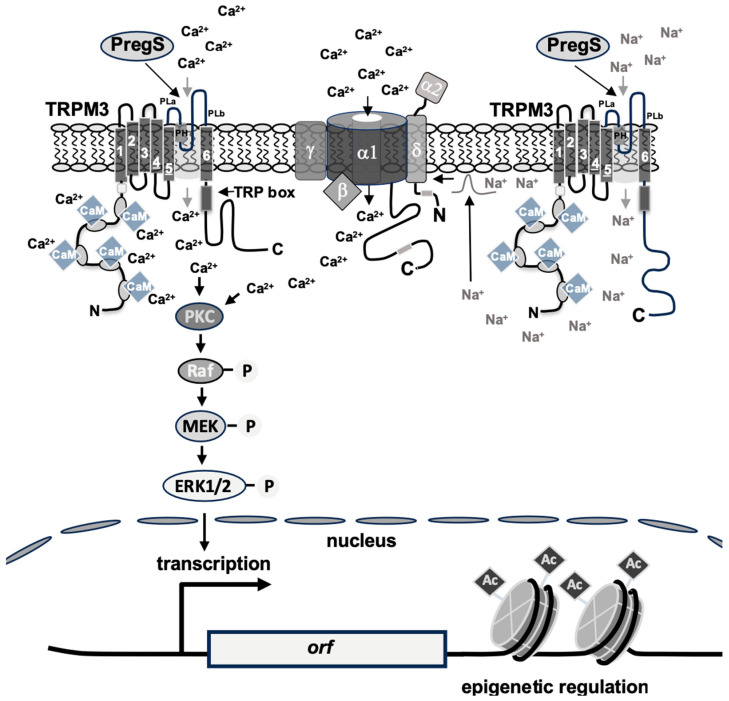
Signaling pathway linking TRPM3 channel stimulation with gene transcription. Stimulation of TRPM3 channels with pregnenolone sulfate (PregS) induces a direct influx of Ca^2+^ ions through the channel into the cells. Alternatively, activated TRPM3 channels can trigger an influx of Na^+^ into the cells, which leads to a depolarization of the plasma membrane and the subsequent activation of voltage-gated Ca^2+^ channels. The result of both scenarios is an increase in the cytosolic Ca^2+^ concentration. This increase in cytoplasmic [Ca^2+^] leads to the activation of protein kinase ERK1/2 via the activation of the protein kinases PKC, Raf, and MEK. ERK1/2 translocates into the nucleus and induces the transcription of stimulus-responsive transcription factors. The transcription factors CREB and Elk-1 are activated via phosphorylation, while the activity of c-Jun and c-Fos activity is increased by both increased transcription and phosphorylation. Activated stimulus-responsive transcription factors regulate the transcription of delayed response genes. Transcription of genes encoding stimulus-responsive transcription factors and delayed response genes is controlled by epigenetic regulators. PH, pore helix; PLa, PLb, poor loop.

In recent years, many important functions of TRPM3 have been identified, including the control of heat sensation and peptide secretion. TRPM3 has been identified as a “pain receptor” [36]. Mutations of TRPM3 have been associated with the development of neuronal disorders and cataracts. TRPM3 channels have been described as a molecular marker of chronic fatigue syndrome/myalgic encephalomyelitis. A major goal of basic research on TRPM3 channels is to understand how stimulation of these channels produces these biochemical and physiological changes. In neurons, TRPM3 stimulation can produce an ionotropic response (Figure 6). The influx of Na^+^ ions facilitates depolarization of the membrane that will propagate the signal by generating action potentials. The influx of Ca^2+^ ions can contribute to the exocytosis of synaptic vesicles. In non-excitable cells, TRPM3 stimulation can cause a metabotropic response (Figure 6). The induction of a signaling cascade leads to the activation of signaling molecules such as protein kinases and transcription factors, which in turn propagates the signal via protein phosphorylation and gene activation of immediate-early and subsequently delayed-response genes. Protein kinase activities of PKC, ERK1/2, and JNK have been linked to mitogenic signaling [88,90], while TRPM3 activity has been correlated with tumorigenesis [15]. Future studies may reveal a causal relationship between mitogenic protein kinases and TRPM3 channel activation. Protein kinase activities of PKC, ERK1/2, and JNK are important signal transducers to the nucleus, where they control gene regulation and transcriptional networks. TRPM3 stimulation leads to the transient activation of stimulus-responsive transcription factors, followed by the transcription of delayed-response genes. The gene products of these delayed response genes are then responsible for the biochemical and physiological changes resulting from TRPM3 stimulation. To date, only a few delayed response genes of TRPM3-mediated signaling have been identified. Interestingly, TRPM3 activates the biosynthesis of three different pro-inflammatory mediators, pointing to a direction for future research on the biological function of TRPM3 channels. One of the future research goals is certainly the identification of more TRPM3-inducible delayed response genes in different tissues.

## Data Availability

No new data were created or analyzed in this study.
